# NE-Motion: Visual Analysis of Stroke Patients Using Motion Sensor Networks

**DOI:** 10.3390/s21134482

**Published:** 2021-06-30

**Authors:** Rodrigo Colnago Contreras, Avinash Parnandi, Bruno Gomes Coelho, Claudio Silva, Heidi Schambra, Luis Gustavo Nonato

**Affiliations:** 1 Department of Applied Mathematics and Statistics, Institute of Mathematics and Computer Sciences, University of São Paulo, São Carlos 13566-590, SP, Brazil; gnonato@icmc.usp.br; 2 School of Medicine, New York University, New York, NY 10017, USA; avinash.parnandi@nyulangone.org (A.P.); heidi.schambra@nyulangone.org (H.S.); 3 Tandon School of Engineering, New York University, New York, NY 10012, USA; bruno.coelho@nyu.edu (B.G.C.); csilva@nyu.edu (C.S.)

**Keywords:** visualization, visual analytics, graph learning, stroke, set theory

## Abstract

A large number of stroke survivors suffer from a significant decrease in upper extremity (UE) function, requiring rehabilitation therapy to boost recovery of UE motion. Assessing the efficacy of treatment strategies is a challenging problem in this context, and is typically accomplished by observing the performance of patients during their execution of daily activities. A more detailed assessment of UE impairment can be undertaken with a clinical bedside test, the UE Fugl–Meyer Assessment, but it fails to examine compensatory movements of functioning body segments that are used to bypass impairment. In this work, we use a graph learning method to build a visualization tool tailored to support the analysis of stroke patients. Called NE-Motion, or Network Environment for Motion Capture Data Analysis, the proposed analytic tool handles a set of time series captured by motion sensors worn by patients so as to enable visual analytic resources to identify abnormalities in movement patterns. Developed in close collaboration with domain experts, NE-Motion is capable of uncovering important phenomena, such as compensation while revealing differences between stroke patients and healthy individuals. The effectiveness of NE-Motion is shown in two case studies designed to analyze particular patients and to compare groups of subjects.

## 1. Introduction

A stroke is a medical condition that results from a prolonged disruption of blood flow to the brain, leading to a loss of function or death [1]. According to Bonita et al. [2], the number of people afflicted by stroke is expected to continue to increase due to demographic changes and inadequate control of risk factors. Therefore, the development of methodologies to assist professionals in the treatment of stroke sequelae is of paramount importance.

The Fugl–Meyer Assessment (FMA) [3] is a clinical bedside instrument to assess abnormal motion in stroke patients. It is considered the gold standard for measuring motor impairment, and is used to guide rehabilitation treatment. The FMA testing protocol evaluates patients as they perform particular movements on both upper extremities (UE), which is the focus of this work, and lower extremities (LE). A trained assessor scores the movements according to a grading scale. The FMA is challenged by the need for a trained assessor and by the subjectivity and coarseness of its grading scale, which both limit the frequent administration of the FMA and the nuanced capture of impairment. In the last decade, FMA scoring has been complemented using data from sensors affixed to the patient’s UE [4,5,6], making it less subjective. These motion sensors generate a large amount of data that can be used to perform tasks other than FMA score computation. For example, the sensor data could be used to generate finer details about movement abnormality and identification of compensatory movements (alternative movement strategies to circumvent impairment). In these cases, the sensor motion data are collected and analyzed from Wearable Sensors Networks (WSNs) [7], which are defined by a set of interconnected sensors dedicated to collecting physiological parameters in the patient’s body. This technology can be used, for example, to diagnose patients through telemedicine applications [6] to analytically detect the compensatory actions of patients in rehabilitation [8], and others [9]. However, computational tools devoted to the visual analysis of a large amount of motion sensor data is quite scarce, especially in the context of assessing the motor function of stroke patients.

In this work, we propose a visual analytic tool to automate the assessment of motor impairment and motor compensation in stroke. Called NE-Motion (Network Environment for Motion Capture Data Analysis), the proposed tool provides resources to visually explore the sensor data and provide greater details on the motion of stroke patients. For example, the proposed tool enables an understanding of the UE segments used by a patient when performing a group of movements and the segments that are more commonly used by stroke patients when performing a particular movement. NE-Motion also enables the comparison of stroke patient and healthy individuals, making it possible to visually identify patients that perform more similarly to healthy individuals. NE-Motion makes use of a graph learning mechanism to transform sensor data into a set of graphs associated to each patient or healthy individual. The graph based representation enables the construction of an elegant and solid mathematical framework to filter out the set of graphs and extract gist information to support the analytical tasks.

In summary, the main contribution of this work are the following:

- A methodology to represent motion sensor data as a set of graphs;
- A mathematical framework to filter the set of graphs so as to extract gist information to support the analytical tasks;
- NE-Motion, a visual analytic tool to assist stroke rehabilitation specialists in identifying impairment patterns and to compare individuals;
- Case studies showing the effectiveness of NE-Motion in answering important questions related to stroke patients.

## 2. Related Work

The literature about visualization methods for sensor data analysis is quite comprehensive. In this section, we focus on visual analytic methods tailored to handle physiological motion data and techniques devoted to the visual analysis and comparison of temporal data. A more comprehensive discussion about fundamental concepts and analytical techniques to deal with WSNs can be found in Khan and Pathan [9] and Mosenia et al. [10] surveys.

### 2.1. Visual Analysis of Motion Capture Data

Motion capture data record body movement over time, typically represented as high-dimensional time-varying information [11]. Application fields where motion capture data play a major role are medicine, sports, games, and animation. In all those fields, the large amount of data involved in the analysis are mostly handled with the aid of visualization systems [12].

Good examples of visualization tools devoted to handling motion capture data include the work by Krekel et al. [13], where a visual widget displays a representation of the upper section of a three-dimensional human skeleton jointly with the angles between limb joints, and Motion Browser [14], which synchronize a patient’s video performing the movement with the visualization of patient’s muscle bundles that have the greatest activation during the movement. Dedicated to visualizing lower extremity motions, the visualization system proposed by Nguyen et al. [15] helps medical professionals to examine patients with patellofemoral instability in the knee, using a radial presentation. Wagner et al. [16] propose KAVAGait, a tool to represent modules of physical forces that act on the human foot during a walk. Motion data are not restricted to human beings. FuryExplorer [17], for example, is a visual analytic tool focused on the analysis of horse motion data, relying on glyph-based silhouettes of horses to describe the position of animals.

Gestures comprise another category of motion data. MotionExplorer [18] groups similar gestures and represents them as evolutions of a stick silhouette enclosed in a circle. GestureAnalyser [19] performs hierarchical and semi-supervised clustering of motions, animating the execution of the movement of each individual. The methodology in Motionflow [20] incorporates a oriented graph to indicate the evolution of a movement. Aiming to detect key positions in the course of movements, Bernard et al. [12] developed a visualization system based on clusters of similar positions derived from classifications.

Table 1 summarizes the main properties of each technique discussed above. Notice that NE-Motion is the only one to handle UE motion data of stroke patients, being also the only one to rely on graph learning to model the problem. In addition, our tool uses visual metaphors dedicated to the visualization of relationships (arc visualization), individual properties (projections), and time series (curve-chart). In contrast to the works described above, we propose a new methodology to represent the “synchronization” between data from the sensors during the execution of specific movements.

### 2.2. Visualization and Comparison of Temporal Data

Motion capture data are a special type of time-varying data [21]. A multitude of visualization tools have been developed to explore time-varying data, ranging from pure time series analysis to multifaceted and graph-based pattern analysis.

In the context of pure time series analysis, there are visualization tools to analyze the growth and decrease of time series [22], methods to explore features in different scales [23], techniques devoted to identify periodicity and anomalies [24], interactive schemes to visually identify patterns [25], among many others. Regarding multifaceted time-varying data, works range from focus + context [26] weather analysis [27,28,29] to multiplayer game analysis [30]. In the context of time-varying data defined on graphs, visualization tools to analyze mobility [31], dynamic graphs [32], and crime data [33] have been proposed. The methods above are only examples of visualization methods for time-varying data and a more comprehensive overview can be found in a number of surveys [21,34,35]

Our approach differs from the methods discussed above in two main aspects: NE-Motion focuses on the synchronization of time series while enabling the visual identification of time series that “synchronize” more often.

## 3. Requirements and Tasks

For almost two years, we interacted with two experts, with an MD and PhD degree, in stroke motion assessment and rehabilitation (AP, HS). From that interaction, we came up with a set of requirements that should be addressed by the analytical tool, which we summarize as follows:

**R1—relation between joint angles:** An important issue when analyzing motion sensor data is to figure out which pairs of joint angles have significant relationships in a given patient and which ones can be disregarded. Such analysis must order the data according to their location in the body.
**R2—prevalent joint angle relationships:** Because of impairment, stroke patients may not move segments of their body that typically move together in healthy individuals. Stroke patients may also use other body segments to circumvent limited motion in impaired body segments. For instance, if a patient cannot flex his shoulder and extend his elbow, he may flex his trunk in order to move his hand forward in space. This means that certain segments are not moving together that normally would in a healthy individual (impairment), while other segments are moving together that normally would not (compensation). Thus, the pairs of sensors that most strongly correlate in stroke patients versus healthy individuals are important to identify.
**R3—focused analysis:** In order to perform certain examinations, experts have to focus on a particular movement, particular individual, and on one side of the body. It is important to be able to easily switch the movement, individual, or body side during the analysis of motions.
**R4—specific patterns:** Identifying patterns that occur only in stroke patients or in healthy individuals is also of great relevance. The identification of individuals with unique behavior (outliers) is also a requirement. Those patterns areuite difficult to get from simple correlation analysis, demanding mechanisms tailored to this end.
**R5—contrast individuals:** A difficulty faced by the experts is the comparison of individuals when an ordinal scale, such as the FMA, is used. Comparing stroke patients according to their impairment level or comparing them against healthy individuals is important to assess the evolution of a rehabilitation process.

We used these requirements to design a set of visualization and analytical tasks for NE-Motion. The mapping among tasks and requirements is indicated in parenthesis:

**Task 1. Overview**: The system must calculate and display the relationships between joint angle data from the sensors, discarding relations that are not of interest. Moreover, the joint angles must be presented in a predefined order according to their location on the body (R1).
**Task 2. Filtering and selection**: The tool should be able to focus the analysis on particular movements, particular individuals, and side of the body (R3). These selection and filtering should be promptly available to the users.
**Task 3. Revealing group-level patterns**: The tool should be able to detect patterns occurring in particular groups of individuals, for example, highlighting relationships between joint angles that are only observed in stroke patients or healthy individuals (R4).
**Task 4. Sorting**: Sorting routines should be implemented to enable the visualization of the most frequent relationships in groups of individuals (R2). This feature will characterize impairment and compensation during motor performance in stroke patients.
**Task 5. Detailing sensor relationships**: Users must be able to visualize detailed joint angle information from the sensors in order to identify trends and extreme values (outliers) (R3 and R4).
**Task 6. Comparing individuals**: The analytical tool should provide mechanisms to compare individuals based on their motion data. This comparison will enable users to identify the individuals that differ most from others. It will identify the data patterns of stroke patients that differ the most from or closely matches healthy individuals and other stroke patients (R5).
**Task 7. Time series presentation**: The tool should allow users to visualize and compare the motion data of a particular participant against an average times series obtained by combining the data from all participants. This feature will enable users to identify typical and spurious behaviors in an individual’s data (R4 and R5).
**Task 8. Global and local analysis**: The system must enable global (group of individuals) as well as local (at the individual level) analysis. Global analysis involves comparing the ’average’ behavior of groups of individuals. In contrast, local analysis enables exploring the performance of a particular individual in a given movement and comparing it against a group of individuals (R3, R4 and R5).

Before providing a match between the tasks above and the design decisions for NE-Motion, we describe the data set provided by our expert partners and the mathematical/computational framework that supports NE-Motion.

## 4. Data Set and Notation

To record the upper extremity (UE) motion, we attached nine inertial measurement unit (IMUs; Noraxon Inc., Scottsdale, AZ) to the upper body (both hands, forearms, arms, spine, and pelvis). Each IMU samples linear acceleration, angular velocity, and magnetic heading at 100 Hz, and the software additionally generates quaternions for each IMU and upper body joint angle values. For the visualization analysis, we used $N_{A}=20$ relevant joint angles at upper body joints (wrist, elbow, shoulder, thoracic spine, and lumbar spine). We used the time series corresponding to the 20 joint angles, described in details in Table 2, for each FMA movement.

UE motion was also recorded synchronously with two cameras (60 FPS, 1088×704; Ninox, Noraxon) positioned orthogonally less than 2 m from the subject. Trained coders used the video data to label the FMA items, which simultaneously labeled the IMU data.

To directly examine the relationship between FMA scores and the output of NE-Motion, each individual performed the FMA while we recorded their motion. We examined $N_{M}=13$ movements of the FMA that entailed arm and wrist motions; the hand items were not examined, given that the IMUs (placed on the back of the hand) did not read out information about the fingers. For data collection, patients are instructed to perform one movement at a time. Specifically, the movements are very simple tasks, such as simulating answering a telephone. There are no repetitions, that is, each individual performs the movement only once. In this case, stroke patients performed the movements on their paretic (affected) side, while healthy individuals performed the movements on both sides of the body. A trained assessor appraised each movement and assigned a score of 0 (for no or incorrect movement), 1 (for partial movement), or 2 (for “normal” movement). Table 3 summarizes the demographic information of the patients in the database. It is noteworthy that this study was carried out in accordance with the recommendations of the Declaration of Helsinki [36,37]. The protocol was approved by the NYU Institutional Review Board.

The data set comprises $N_{P}^{{\mathrm{stroke}}_{L}}=28$ of left-paretic stroke patients (impaired on the left side of the body), $N_{P}^{{\mathrm{stroke}}_{R}}=23$ of right-paretic stroke patients, and $N_{P}^{{\mathrm{CRTL}}_{R}}=N_{P}^{{\mathrm{CRTL}}_{L}}=18$ of healthy individuals, totaling $N_{P}=N_{P}^{\mathrm{stroke}}+N_{P}^{\mathrm{CRTL}}=N_{P}^{{\mathrm{stroke}}_{R}}+N_{P}^{{\mathrm{stroke}}_{L}}+N_{P}^{{\mathrm{CRTL}}_{R}}+N_{P}^{{\mathrm{CRTL}}_{L}}=87$ individuals. We use $N_{A}=20$ joint angles. Therefore, the data set contains $N_{T}$ time series, where $N_{T}$ is given by the following:

(1) $$N_{T}=N_{P}\cdot N_{M}\cdot N_{A}=87\cdot 13\cdot 20=22620.$$

To settle the notation, consider the following sets:

- Joint Angles: $A:=\left\{a_{1},a_{2},...,a_{N_{A}}\right\};$
- Movements: $M:=\left\{M_{1},M_{2},...,M_{N_{M}}\right\};$
- Participants: $P:=\underset{P\mathrm{stroke}}{\underset{︸}{\{p_{1},p_{2},...,p_{N_{P}^{\mathrm{stroke}}}}},\underset{P\mathrm{CTRL}}{\underset{︸}{p_{N_{P}^{\mathrm{stroke}}+1},...,p_{N_{P}}\}}}$,

where $P^{\mathrm{stroke}}$ is the set of stroke patients and $P^{\mathrm{CTRL}}$ is the set of healthy controls. We divide the sets according to the side of the body that has been recorded, that is, the following:

○ $P^{\mathrm{stroke}}:=P^{{\mathrm{stroke}}_{L}}\cup P^{{\mathrm{stroke}}_{R}}$,
○ $P^{\mathrm{CTRL}}:=P^{{\mathrm{CTRL}}_{L}}\cup P^{{\mathrm{CTRL}}_{L}}$;

The set of time series T is denoted by:

- Time series: $T=\underset{p_{i}\in P,M_{j}\in M}{⋃}T_{i,j}$,

where $T_{i,j}:=\left\{t_{i,j,1},t_{i,j,2},...,t_{i,j,N_{A}}\right\}$ accounts for the set of time series of the individual $p_{i}$ performing the movement $M_{j}$.

A main issue to understanding how each individual or groups of individuals behave is to figure out which pairs of joint angles “correlate” (synchronize) during the movements accomplished by the individuals, as such correlations point out which parts of the body are concurrently moving during the movement. In the next section, we present the mathematical foundation of the methodology we developed to tackle the analysis of the movements and individuals.

As each individual may take a different amount of time to perform the same movement, the number of time steps of each time series in T may be different. Thus, it is necessary to transfer all time series of T to a space of the same dimension. In this case, we perform a coordinate up-sampling procedure using spline polynomials [38], so that we transfer all time series of T to the space of dimension $m_{\max}$, with $m_{\max}$ being of length 1500 since 98.8% of the data has a size equal or lower than this.

Therefore, we will consider all time series as having the same number of time steps.

## 5. Mathematical Foundation

The proposed methodology relies on Graph Learning (GL) theory [39] to identify and represent the relation between pairs of joint angles. An undirected network, or a graph, G(V,E) is given by a set of vertices $V=\{v_{1},v_{2},...,v_{n}\}$ and a set of edges E⊂V×V, where $(v_{i},v_{j})\in E$ if the vertices $v_{i}$ and $v_{j}$ are linked in *G*. The graph *G* can be represented by an adjacency matrix $W\in R^{n\times n}$, where an entry $W_{i,j}$ in *W* is non-zero if and only if $(v_{i},v_{j})\in E$. Since *G* is undirected, the matrix *W* is symmetric.

The GL problem consists of defining the edge set *E* from a set of vertices *V* and multivariate data *X* associated to each vertex $v_{i}\in V$. In other words, in GL, the set of edges *E*, and thus *W*, are unknown and must be computed from *V* and *X*. Specifically, the multivariate data *X* can be represented as a matrix $X={\left[x_{1},x_{2},...,x_{n}\right]}^{T}\in R^{n\times m}$, where the row $x_{i}\in R^{m}$ corresponds to a vector associated with the vertex $v_{i}\in V$ of *G*.

To compute the set of edges *E*, GL techniques assume certain premises, being that “smoothness” is one of the most used hypotheses. Intuitively, smoothness seeks to ensure that a pair of vertices $v_{i}$ and $v_{j}$ are connected by an edge $(v_{i},v_{j})\in E$ if the vectors $x_{i}$ and $x_{j}$ associated to those vertices are similar to each other, that is, $∥x_{i}-x_{j}∥$ must be close to zero. In other words, the edge set *E* should connect the most similar vertices. In mathematical terms, the resulting edge set must give rise to an adjacency matrix *W* that minimizes the Dirichlet energy [40].

(2) $$D_{W}\left(X\right):=\frac{1}{2}\sum_{i=1}^{n}\sum_{j=1}^{n}W_{i,j}{∥x_{i}-x_{j}∥}^{2}.$$

Equation (2) tells that $W_{i,j}$ must be zero when $∥x_{i}-x_{j}∥$ is large. Moreover, large values of $W_{i,j}$ correspond to edges connecting quite similar vertices ($∥x_{i}-x_{j}∥$ close to zero).

To compute an adjacency matrix *W* (and thus the edge set *E*) that minimizes Equation (2) while ensuring that *W* is symmetric, non-negative, and sparse, we rely on the optimization procedure proposed by Kalofolias [41], which consists of solving the following mathematical problem:

(3) $$\begin{array}{cccc} W^{*} & := & \arg\min & D_{W}\left(X\right)+g\left(W\right).\\ \; & \; & W\in W \end{array}$$

where $W=\{W\in R^{n\times n}\;|\;W=W^{T},W_{i,i}=0,\mathrm{and}W_{i,j}\geq 0,\forall i,j\}$. The right most regularization term g(W) aims to ensure sparsity and connectness and it is defined as follows:

(4) $$g\left(W\right)=-\alpha \left(1^{T}\cdot \log\left(W\cdot 1\right)\right)+\gamma \cdot {∥W∥}_{F}^{2},$$

where $1\in R^{n}$ is a vector with all coordinates equal to 1, α>0 and γ≥0 are parameters set as proposed by Kalofolias and Perraudin [42]. The logarithmic term is applied in each entry of the vector W·1 and it enforces each vertex to be connected to other vertices of the graph since an isolated vertex result in row with zeros in *W*. The second term ${∥W∥}_{F}^{2}$ avoids the trivial solution while enforcing sparsity.

Kalofolias [41] demonstrates in his work that the framework derived from Equations (3) and (4) can be modeled to be solved by the primal–dual scalar optimization procedure [43] that is scalable while presenting fast convergence, making this methodology doable even for large scale problems. Specifically, as $D_{W}+g$ is a lower semi-continuous convex function [44], the optimization problem discussed here is guaranteed to converge to the minimum in a very efficient way.

In the following section, we show how we use GL to build networks whose edges represent the relation between pairs of joint angles.

## 6. Network Construction

**Figure 1:**
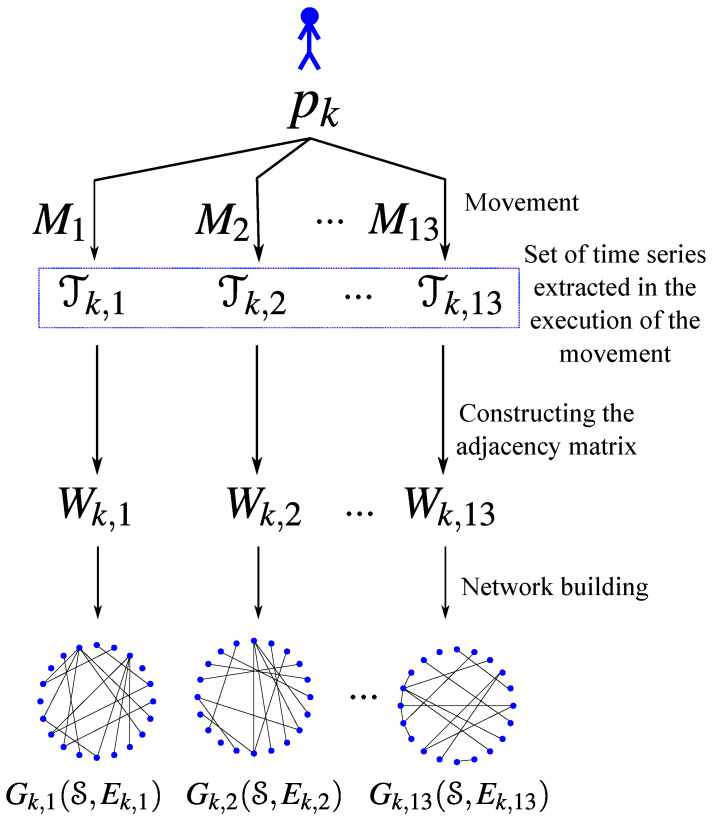
Construction of the networks associated to an individual $p_{k}$. One network for each movement.

We rely on the GL methodology discussed in Section 5 to build a network for each movement. Therefore, each individual $p_{k}\in P$ has a set of associated networks, one for each movement $M_{l}\in M$. The vertices of each network correspond to the joint angles, so all networks have the same number of vertices. Each vertex (joint angle) has a time series associated to it and the time series play the role of the multivariate data $x_{i}$ discussed in Section 5. Given the vertices and the time series associated with them, we compute the edges of the network associated with a movement by solving the GL problem (3). Since the GL results in the edges connecting similar nodes, the edges naturally indicate the pairs of joint angles that most correlate during a movement. Figure 1 illustrates the process we use to compute the set of networks associated with each individual.

As pointed out in requirement R1, some correlations between joint angles are of no interest, so they must not be represented as edges in the network. Specifically, null time series and time series with nearly constant values are of no interest, as those time series are not recording a movement. To avoid edges connecting vertices corresponding to joint angles whose time series are null and nearly constant, we modify the similarity measured (see Equation (2)) to become the following:

(5) $$D_{W}\left(X\right):=\frac{1}{2}\sum_{i=1}^{n}\sum_{j=1}^{n}W_{i,j}Z_{i,j},$$

where $Z_{i,j}$ is given by

(6) $$Z_{i,j}:=\alpha z_{1}\left(i,j\right)+\beta z_{2}\left(i,j\right)+\gamma z_{3}\left(i,j\right).$$

where $z_{1}\left(i,j\right)={∥t_{k,l,i}-t_{k,l,j}∥}^{2}$ and $t_{k,l,i}$ accounts for the time series associated to joint angle $a_{i}$ of the individual $p_{k}$ performing the movement $M_{l}$. The values α,β, and γ are non-negative parameters that control the importance of each term in $Z_{i,j}$. In this work, we define (α,β,γ)=(1,1,1). The term $z_{2}\left(i,j\right)$ penalizes the relations between null time series and it is given by the following:

(7) $$z_{2}\left(i,j\right):=\left\{\begin{array}{cc} 2, & \mathrm{if}\;t_{k,l,i}\;\mathrm{or}\;t_{k,l,j}\mathrm{are}\;\mathrm{null},\\ 0, & \mathrm{otherwise}. \end{array}\right.$$

The term $z_{3}\left(i,j\right)$ in Equation (6) penalizes constant time series and is defined as follows:

(8) $$z_{3}\left(i,j\right):=e^{-\mathrm{diff}(t_{k,l,i})}+e^{-\mathrm{diff}(t_{k,l,j})},$$

where diff is a function that calculates the sum of the absolute value of the differences between consecutive time series values, that is, the following:

(9) $$\mathrm{diff}\left(x\right):=\sum_{i=1}^{m-1}\left|x_{i}-x_{i+1}\right|,\;\;x\in R^{m}.$$

Algorithm 1 summarizes the steps for creating a graph $G_{k,l}$ associated with the individual $p_{k}\in P$ and movement $M_{l}$. The algorithm takes as input the time series $T_{k,l}$ representing the respective joint angles and it outputs the networks $G_{k,l}\left(S,E_{k,l}\right)$, that is, the adjacency matrices $W_{k,l}={\left[{\left(W_{k,l}\right)}_{i,j}\right]}_{i,j=1}^{N_{A}}$. In terms of computational times, the algorithm takes around a tenth of a second to calculate one network and, consequently, as we need to calculate 87·13=1131 networks, the method takes around 113.1 s to compute all the networks in a personal computer with 2.4 GHz Intel(R) Core i7 CPU and 16 GB of RAM.
**Algorithm 1** Computing a network.1:**Input:**$T_{k,l}=\left\{t_{k,l,1},t_{k,l,2},...,t_{k,l,N_{A}}\right\}$: set of $N_{A}$ time series representing the joint angles of individual $p_{k}$ when performing the movement $M_{l}$.2:**Output:**$W_{k,l}$: adjacency matrix corresponding to the network $G_{k,l}\left(S,E_{k,l}\right)$.3:Z:=0                    ▹$0\in R^{N_{A}\times N_{A}}$ is the null matrix4:**for**i:=1 to $N_{A}$ **do**5:    **for** j:=i+1 to $N_{A}$ **do**6:        $Z_{i,j}:=z_{1}\left(i,j\right)+z_{2}\left(i,j\right)+z_{3}\left(i,j\right)$7:        $Z_{i,j}:=Z_{j,i}$8:    **end for**9:**end for**10:Solve the optimization problem in the Equation (3), given the pairwise similarity measures $Z={\left(Z_{i,j}\right)}_{i,j=1}^{N_{A}}$, to obtain the adjacency matrix $W_{k,l}$.

### 6.1. Filtering Networks

In order to accomplish the tasks described in Section 3, the networks associated to an individual or group of individuals must be filtered out to get the information of interest. For instance, if the focus of the analysis is on movement $M_{l}$ performed by patients with stroke on the left side of the body, the filtering mechanism should return the set of networks associated with that movement for that group of individuals.

We rely on set theory [45] as the building block for the design of a family of filters that support our tasks. Let $G_{U}$ denote a graph-like structure where the nodes correspond to the joint angles, but the edges are the union of the edges from all networks, that is, the following:

(10) $$G_{U}=G_{U}\left(A,E_{U}\right)=G_{U}\left(A,\underset{p_{k}\in P,M_{l}\in M}{⋃}E_{k,l}\right).$$

where A is the vertex set corresponding to joint angles and $E_{U}$ comprises the set of all edges from all networks. Mathematically, $G_{U}$ is not a graph in the classical sense since it may have several edges connecting the same pairs of vertices; however, the structure of $G_{U}$ will serve our purposes. We denote by Λ a filter that extracts from $E_{U}$ the subset of edges that must be involved in a given analysis. For instance, suppose that the domain of interest is stroke patients affected on the left side of the body ($P^{{\mathrm{stroke}}_{L}}$) and movement $M_{2}$ (l=2). Thus, the filter Λ must be the following:

(11) $$\Lambda =\left\{\begin{array}{c} \prime \prime \mathrm{stroke}\;\mathrm{individuals}\\ \mathrm{movement}M_{2}\\ \mathrm{left}\;\mathrm{side}\prime \prime \end{array}\right\}.$$

Mathematically, the filter is implemented as follows:

(12) $$E_{\Lambda }=\left\{e\in ⋃E_{k,l}\subset E_{U}\;|\;p_{k}\in P^{{\mathrm{stroke}}_{L}},\;\;l=2\right\},$$

giving rise to

(13) $$G_{\Lambda }=G_{\Lambda }\left(A,E_{\Lambda }\right).$$

#### Filtering Edge Functions

Certain tasks demand that edges from $E_{U}$ are filtered based on the values of a function defined on $E_{U}$. Suppose, for example, that one wants to figure out which is the edge that is more prevalent in the networks of healthy individuals when performing the movement $M_{8}$ with the right side of the body. In this case, the filter Λ can be stated as follows:

(14) $$\Lambda =\left\{\begin{array}{c} \prime \prime \mathrm{most}\;\mathrm{prevalent}\;\mathrm{edge}\\ \mathrm{healthy}\;\mathrm{individuals}\\ \mathrm{movement}\;M_{8}\\ \mathrm{right}\;\mathrm{side}\;\mathrm{of}\;\mathrm{the}\;\mathrm{body}\prime \prime \end{array}\right\}.$$

To accomplish the filtering we define the function $f_{\mathrm{count}}:E_{U}\to R$ as follows:

(15) $$f_{\mathrm{count}}\left(e\right):=\#\left(\left\{E_{k,l}\subset E_{U}\;|\begin{array}{c} le\in E_{k,l},\\ p_{k}\in P^{{\mathrm{CTRL}}_{R}},\\ l=8 \end{array}\right\}\right),$$

where #(·) is the number of elements in a set.

The filtered subset of edges $E_{\Lambda }$ is obtained as follows:

(16) $$E_{\Lambda }=\left\{e\in ⋃E_{k,l}\;|\begin{array}{c} p_{k}\in P^{{\mathrm{CTRL}}_{R}},\\ l=8,\\ f_{\mathrm{count}}\left(e\right)=\Gamma \left(e\right) \end{array}\right\},$$

where $\Gamma \left(e\right):=\max\left\{f_{\mathrm{count}}\left(e\right)\;|\;e\in ⋃E_{k,l}\right\}$.

The filtering scheme presented above provides a unified manner of filtering edges in order to accomplish local and global analysis involved in the tasks described in Section 3.

## 7. NE-Motion

NE-Motion is a visual analytic system designed to tackle the tasks outlined in Section 3. Each of the visual components described in the following were designed based on thorough discussions with the experts, where the pros and cons of each possible alternative were analyzed until consensus was reached. Several adjustments were made during the course of development to tackle issues and to improve the analytical capability of the proposed visualization tool.

**Figure 2:**
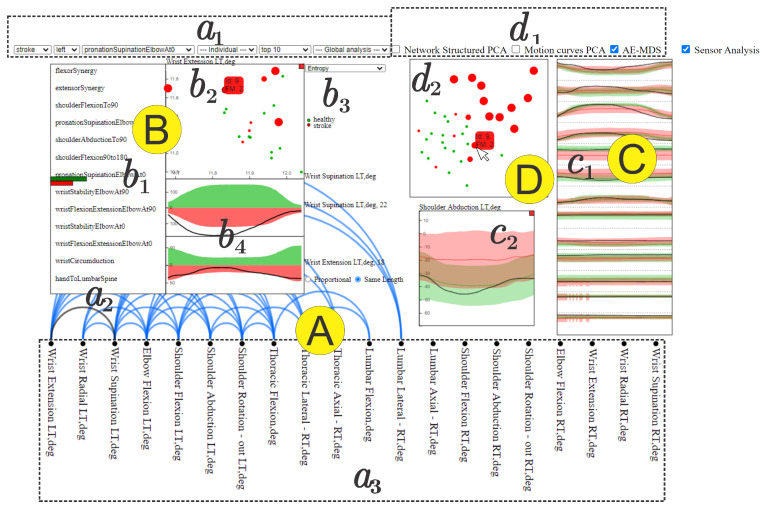
NE-Motion visualization tool. Component **A** is responsible for presenting the pairwise relation ($a_{2}$) between joint angles ($a_{3}$) resulting from a filtering, which is interactively specified by filtering menu ($a_{1}$). The visualization component **B** details the result of the filter and enables the comparison of movements while providing an overview of the time series. In this case, $b_{1}$ graphically represents the numerical ratio between healthy control (green bar) and stroke patients (red bar); $b_{2}$ is a scatter plot of the statistical measure defined in $b_{3}$ extracted from the time series of each joint angle; in $b_{4}$, a mirrored graph is shown between average healthy control motion curves (at the top in green) and stroke patients (at the bottom in red) for each joint angle that makes up the selected edge. Component **C** shows the statistical summary extracted from the filtered time series associated with the joint angles, also allowing a visual comparison of a particular individual against the summary. While, in $c_{1}$, we see an overview that takes into account all the joint angles that have some synchronization between them, in $c_{2}$, we see in detail the summary of only one joint angle at a time. Component **D** enables the comparison of the individuals through projections, defined in menu ($d_{1}$), and presented in an interactive chart ($d_{2}$).

Figure 2 shows the main components of NE-Motion, which are detailed in the following.

**Filtering Menu.** The filtering menu (component $a_{1}$ in Figure 2) enables users to interactively define a filter Λ, thus addressing tasks $T_{2}$ and $T_{4}$ while supporting the accomplishment of task $T_{3}$. Users can select the following:

- A particular category of individuals: stroke, healthy, or both.
- Side of the body: left or right.
- Choose a particular individual.
- Sort by top *K* most prevalent relations.
- Edges that take place concurrently on both stroke patients and healthy control individuals or that appear in one category but are not present in the other.

The available filtering options enable many such different analyses as, for example, analyzing the similarities and differences between healthy and stroke affected individuals.

**Pairwise Relationship View** NE-Motion relies on an arc visualization metaphor [46] to reveal the relation of joint angles resulting from a filtering (edges in $E_{\Lambda }$). The joint angles (Figure 2 $a_{3}$) are arranged according to a pre-established order determined by the specialists and pairs of joint angles containing edges in $E_{\Lambda }$ are connected by an arc (Figure 2 $a_{2}$). Users can interactively select a particular arc (relation) to get details about the group of individuals where the selected relationship is present. This component addresses task $T_{1}$ and support task $T_{5}$.

**Detailed Relation View** This component, illustrated in Figure 2*B*, is responsible for highlighting details about the relationship of a pair of joint angles. The *detailed relation view* is triggered when the user selects a particular arc in the *pairwise relations view* and it shows the proportion of healthy control and stroke patients that present that relationship in each movement selected by the user (Figure 2 $b_{1}$). A scatter plot (Figure 2 $b_{2}$) derived from measurements computed from the time series associated the each joint angle linked by the selected arc is also provided. Specifically, each axis in the scatter plot corresponds to a joint angle; each point represents an individual (green is healthy and red is stroke), and the coordinates of the points are given by the measurement extracted from the time series of the individuals. Users can choose (Figure 2 $b_{3}$) three different measurements: mean value, entropy, and energy of the time series. In addition, the size of each circle is defined according to the FMA score of the represented individual in which the larger the circle, the lower the FMA score and, therefore, the more impaired the motion. The visualization widget shown in Figure 2 $b_{4}$ shows the average shape of the time series associated with the pair of joint angles linked by the selected arc. The green time series correspond to healthy individuals and the red ones to stroke patients, as requested by the domain experts. In this case, the average time series of stroke patients is mirrored on the *y*-axis, enabling the user to compare the kinematic profiles of the joint angle changes over time. In addition, for $b_{4}$, the tool provides the user with two viewing options: “Proportional” and “Same Length”. The “Proportional” view displays the average time steps of each group’s time series, which may be shorter in healthy subjects and longer in stroke patients. The “Same Length” view displays the time series of both groups in same number of time-steps, enabling the direct comparison of the kinematic profiles. If an individual is selected in the scatter plot by hovering the mouse on the point, the particular time series of that individual is also presented (black curve in the plot), making it possible to compare the individual against the group average. The detailed relation view addresses tasks $T_{3},T_{5},T_{6}$ and $T_{7}$ while supporting $T_{8}$.

**Figure 3:**
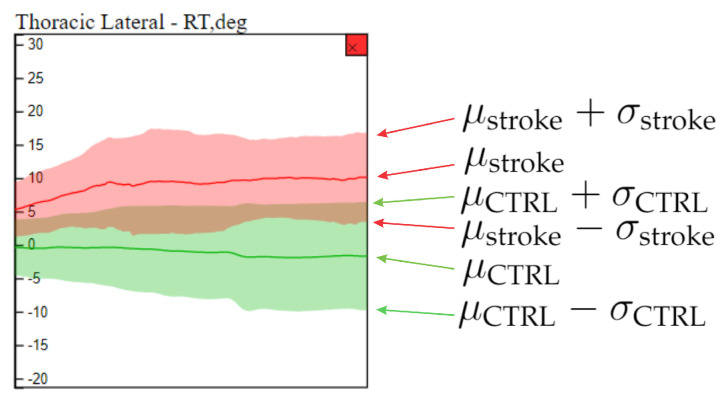
Statistical curve based summary of the time series behavior of stroke patients and healthy control individuals in a particular joint angle (thoracic lateral). The green and red curves refer to healthy control and stroke patients respectively. The average ($\mu_{\mathrm{CTRL}}$ and $\mu_{\mathrm{stroke}})$ and standard deviation ($\sigma_{\mathrm{CTRL}}$ and $\sigma_{\mathrm{stroke}}$) curves summarize the behavior of each group of individuals.

**Comparison View** This analytical component provides resources to compare individuals in the whole data set. It comprises three main visual widgets. The first widget, illustrated in Figure 2 $c_{1}$, presents the average and standard deviation curves for the time series associated to each joint angle for both healthy (green) and stroke individuals (red). The time series are ordered in this component according to the joint angle arrangement of component $a_{3}$, that is, the box topper in $c_{1}$ refers to the leftmost joint angle in $a_{3}$. In this case, the time series from the whole set of movements can be analyzed or filtered based on a particular movement (one side of the body must be chosen). When a particular joint angle is selected, a detailed view of that joint angle shows up, as illustrated in Figure 2 $c_{2}$, allowing to visually compare, through a statistical curve based summary, the time series behavior of stroke patients and healthy control individuals in the chosen joint angle. Figure 3 shows the elements in the detailed view.

In order to provide an overview on how individuals compare to each other, the comparison view enables a multidimensional projection visual widget (Figure 2*D*). Three different projection schemes are provided (Figure 2 $d_{1}$): network principal component analysis (PCA), motion curves PCA, and auto-encoder multidimensional scaling (AE-MDS).

The network PCA maps to a 2D space a multidimensional feature vector derived from the set of networks of each individual. Specifically, for each network we extract the following measures: node degree ($f_{\deg}$) [47], closeness centrality ($f_{\mathrm{close}}$) [48]; eigencentrality ($f_{\mathrm{eig}}$) [49], and clustering coefficient ($f_{\mathrm{clus}}$) [50]. Therefore, for each movement, we have a feature vector in $R^{4\cdot N_{A}}$. If all movements are considered simultaneously, the feature vector is $N_{M}\left(4N_{A}\right)$-dimensional.

Motion curves PCA simply consider each normalized time series as high-dimensional data and project the curves in a two-dimensional space using PCA.

AE-MDS projection uses MDS to project an embedding of each individual from a multidimensional space. The embedding is accomplished, using an autoencoder trained with all time series. To increase the amount of training data, we also included the time series in reversed order in the training data. The encoder architecture consists of six layers of 1D convolutions. The first convolutional layer transforms the input to four channels while maintaining its length. The next five layers halve the length and the amount of channels, reaching a dimension of 188, which we use as the embedded representation for each individual. The decoder has a similar architecture to the encoder, except that in the encoder, we use a Relu activation function, while in the decoder, no activation is used. PCA was chosen for projecting network structures and motion curves because it handles high-dimensional data quite well while being computationally efficient. Since the AE embeds the curves in a relatively low dimensional space, MDS can be used, providing an alternative visualization.

An individual can be selected in the projection layout (Figure 2 $d_{2}$) by hovering the mouse on the corresponding point (the ID of the individual shows up by hovering the mouse). Once selected, the time series associated with that individual are shown in the detailed view. The comparison view was designed to address task $T_{6},T_{7}$ and $T_{8}$.

All NE-Motion visual components can be dragged over the screen for repositioning, facilitating the visualization and comparison of the displayed information.

### Implementation

The network construction is done using PyGSP [51] in Python. Time series are stored in a database using MySQL and queried through PHP and Ajax. NE-Motion visual components are coded in JavaScript and D3.js. The autoencoder is built using TensorFlow.

## 8. Case Studies

In this section, we present two case studies carried out jointly with the experts that closely collaborated in the development of NE-Motion. The first case study focuses on the visual identification of patterns and comparison between individuals. The second case study shows the utility of NE-Motion to enable a detailed analysis of stroke patients so as to reveal the similarities in their motion.

### 8.1. Case Study I: Visual Identification of Patterns and Comparison between Individuals

In this case study, we focus on **Comparison View** and **Pairwise Relations View** NE-Motion components to visually identify patterns and compare individuals. Specifically, from the **Filtering menu** we select the movement “flexor synergy” and the left side of the body (Task $T_{2}$). Starting with their hand on their knee, the flexor synergy item of the FMA requires the individual to raise their hand toward the ear, bringing the elbow up to shoulder height with the thumb pointed toward the ceiling as if they are answering a telephone.

**Figure 4:**
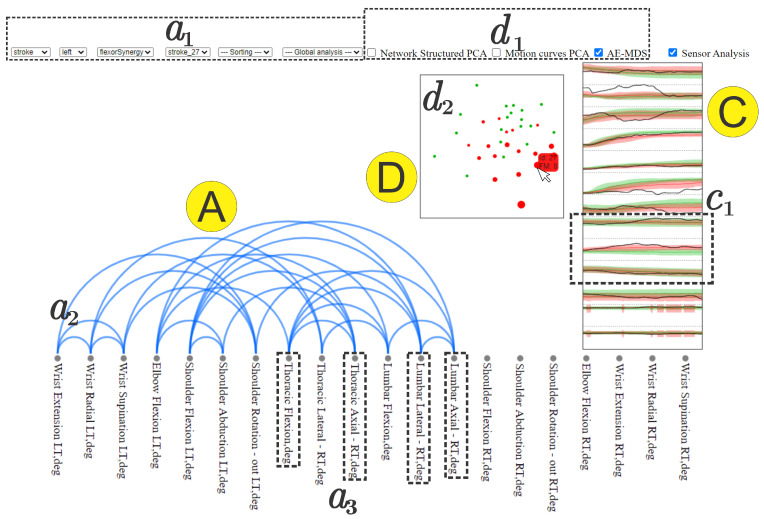
Using NE-Motion to identify patterns and analyze particular individuals. We use components **A**, **C**, and **D**, discussed in Figure 2. In $a_{1}$, we defined the domain of interest: the individual “stroke_27” and the flexor synergy movement performed with the left side of the body. In the right of the image (component **C**), we can see the time series (mean and standard deviation curves) that describe the UE joint angles of healthy (green curves) and stroke (red curves) individuals who performed exercise “flexor synergy” with the left side of the body, representing a global analysis. In the center, plot ($d_{2}$) shows the MDS projection of the deep features extracted with an autoencoder, indicated as AE-MDS on $d_{1}$, from the time series of healthy individuals (green circle) and stroke individuals (red circle), representing the local analysis. The tool allows the user to select an individual through mouse hover event, which triggers, in the second resource, the display of the time series in $c_{1}$ (in black) of all the joint angles of the selected individual and also shows in $d_{2}$ a tag with his/her FMA score for the movement. The dashed rectangles in the bottom ($a_{3}$ component) highlight nodes in the **Pairwise Relations View** component that have a large number of connections ($a_{2}$). The dashed rectangle on the right ($c_{1}$) highlights truncal motion curves that can be associated with compensatory motion in lumbar and thoracic spine.

To perform the comparison between individuals, we trigger two NE-Motion visual components: the pairwise relations view and the comparison view as shown, respectively, in Figure 4-*A* and C/D. In this case, for the AE-MDS projection ($d_{2}$), the color of the circles refers to the subject group (red for stroke patients and green for healthy individuals) and the radii of the circles indicate the impairment level (circles with larger radii refer to higher impairment). The projection shows that there is a segregation between the groups with stroke patients (red circles) concentrated on the bottom right of the projection, and healthy individuals (green circles) concentrated on top of the projection layout (Task $T_{6}$). We also note that the projection corresponds well with the FMA scores: stroke patients with high FMA scores (i.e., less impairment) are projected close to the healthy cluster in the AE-MDS projection.

In contrast, stroke patients with lower FMA scores (larger diameter) are projected away from the healthy cluster. This indicates that our model, based on an AE neural network, used to embed the joint angle time series was able to properly group individuals according to their impairment level. Consequently, we can note that the projection is able to capture the differences in the movements performed by stroke patients and healthy control individuals. This analysis attests the effectiveness of NE-Motion mathematical and analytical design, making possible to represent individuals with similar level of impairment in an intuitive manner.

**Figure 5:**
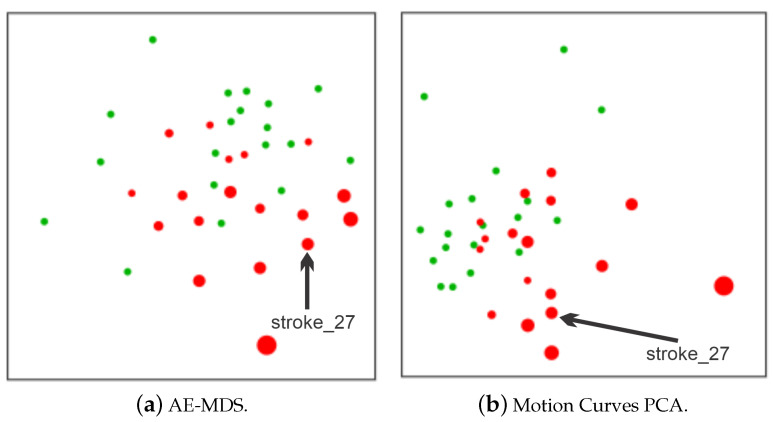
Using two different projection schemes ((**a**) AE-MDS and (**b**) PCA) in the **Comparison View** component to assess how close stroke patients are to the healthy individuals. All individuals who performed the “flexor synergy” movement are represented in both projections, with green circles representing healthy and red circles representing stroke. The larger the diameter of a red circle, the lower the individual’s FMA score, indicating more impairment. In both projections, we can note good separation between the group of control individuals and stroke patients who performed the movement with less difficulty (smaller circles) from the group formed by stroke patients with more difficulty in movement (larger circles). In addition, the individual “stroke_27” is represented close to patients with a similar level of impairment to FMA scores.

Directing our analysis on the individual “stroke_27” (score 8 out of 12 on flexor synergy item and a total score 47 out of 66 on the FMA), we can see in the **Comparison View** component in Figure 4 that “stroke_27” is projected near other stroke individuals with similar diameters (i.e., similar FMA scores). This trend is observed in both AE-MDS and PCA projection, which demonstrates that the motion data of each group of individuals have well-defined features within each category and that the patient in question has similar time series to other patients with a similar FMA score, as shown in Figure 5.

**Figure 6:**
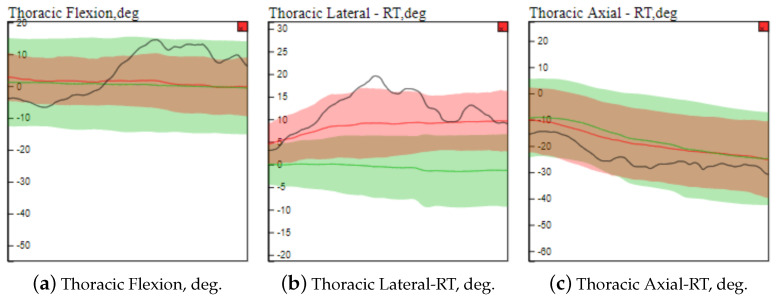
Visualizing information about the motion curves of three thoracic joint angles ((**a**) thoracic flexion, (**b**) thoracic lateral, and (**c**) thoracic axial) using the **Comparison View** zoom resource. In this case, NE-Motion considers the respective motion curves of all individuals who performed the flexor synergy movement with the left side. Average motion curves are represented by the red line (stroke individuals) and the green line (healthy individuals). The area around these average curves is defined by the distance between them and the standard deviation of the motion curves for each category of individuals considered. The black curve is the motion curves of the “stroke_27” patient. We can notice that the motion curves of the analyzed individual do not present smoothness and are very different from average curves in all cases. Specifically, in (**b**), we see that the joint angle assumes values even outside the standard deviation of the affected individual motion curves. This can serve as an indication for intense compensatory actions of the patient.

In addition, when analyzing the motion time series for “stroke_27” in the **Comparison View** component in Figure 4-*C* and, more in-depth, in the zoomed view depicted in Figure 6, it is evident that this subject’s joint angle motion curves (shown as black lines) are not smooth and, in some cases, are outside the statistical limits relative to the curves of the other individuals with similar condition (tasks $T_{6}$, $T_{7}$ and $T_{8}$).

Indeed, if we look at its joint angle network presented in the **Pairwise Relations View** component (task $T_{1}$), nodes in the thoracic and lumbar regions have a high degree of connectivity with various nodes of the upper body. This connectivity indicates that the truncal motion is associated with limb motion, as highlighted by the bottom dashed rectangles in Figure 4-$a_{2}$ and $a_{3}$.

Focusing on the trunk (curves highlighted with the dashed rectangle in Figure 4-*c1* and broken out in Figure 6), we can see that for “stroke_27”, the motion of the thoracic spine is more pronounced and different from other individuals. This is an indication that this individual may be using compensatory motions during completion of the “flexor synergy” FMA item.

### 8.2. Case Study II: Understanding Patterns of Stroke Patients

This second case study focused on exploring the features belonging only to stroke individuals. To this end, from the **Filtering menu** we selected the FMA item “Shoulder Flexion $90^{\circ }$ to $180^{\circ }$” on the left side of the body. In this movement, the subject starts with their arm held out in front of them (shoulder flexed to $90^{\circ }$ and elbow extended to $0^{\circ }$) and lifts their arm up (shoulder flexed to $180^{\circ }$ and elbow extended to $0^{\circ }$). Subjects were instructed to keep their elbow straight at all times during the movement.

**Figure 7:**
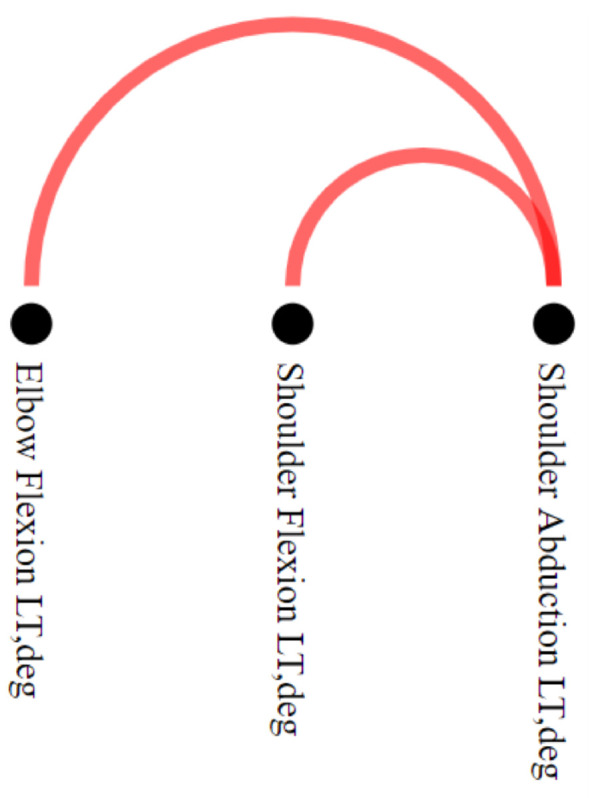
Use of **Pairwise Relations View** in “Differences” special filtering, which uses different colors to represent the edges that are only present in stroke patients (red) or healthy individuals (green). In the case study, comparing the data generated considering “Shoulder Flexion $90^{\circ }$ to $180^{\circ }$” for the left side, only the red edges between elbow flexion and shoulder abduction, and shoulder flexion and shoulder abduction persisted. These edges are not present in any network of healthy individuals, indicating that the synchronizations in question may be associated with abnormalities in movement.

To explore the differences between stroke patients and healthy control groups, we filtered out the edges that simultaneously appeared in networks associated with both groups. This filtering can be performed by setting the option “Differences” in the **Filtering menu**. The filtering operation resulted in a motion graph with only two connections concerning stroke individuals and none for healthy individuals (task $T_{3}$), as we can see in the NE-Motion **Pairwise Relations View** component presented in Figure 7. Inother words, NE-Motion shows that only two joint angle synchronizations, unique to stroke patients, occur during the execution of the movement “Shoulder Flexion $90^{\circ }$ to $180^{\circ }$” with the left side. In this case, we can see that these synchronizations take place between the joint angles “Shoulder Abduction LT, deg” and “Shoulder Flexion LT, deg” and “Shoulder Abduction LT, deg” and “Elbow Flexion LT, deg”.

**Figure 8:**
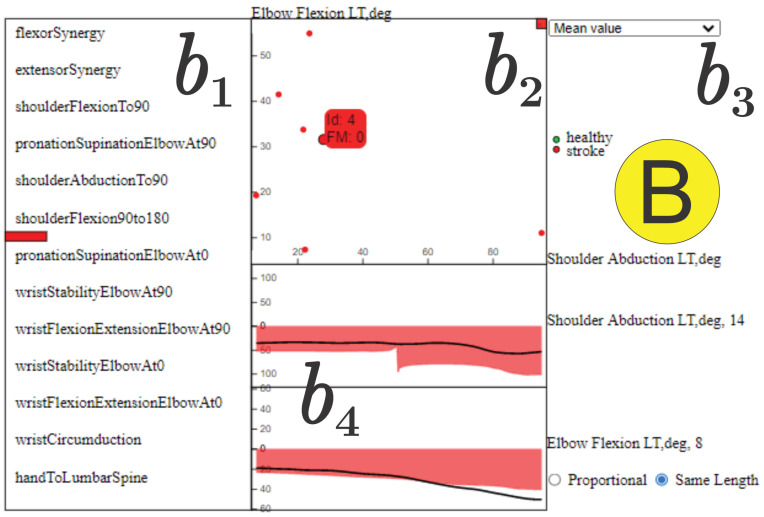
**Detailed Relation View** showing a detailed view of the time series associated to joint angles, “Shoulder Abduction” and “Elbow Flexion”. The black curve in $b_{4}$ shows the data from one stroke individual (“stroke_4”) performing the “Shoulder Flexion $90^{\circ }$ to $180^{\circ }$”. In the component, there are seven stroke individuals ($b_{1}$ and $b_{2}$) for which these joint angles are significantly synchronized, and the average motion curve of these individuals is shown in $b_{4}$ for each of these joint angles as a red area mirrored on the *y* axis. This shows that “Shoulder Abduction” and “Elbow Flexion” are synchronized, suggesting the intrusion of flexor synergy. Note the absence of healthy control information (green circles and green curve) for this edge, indicating that synchronous activity in these joint angles does not normally occur in healthy movement.

Activating the **Detailed Relation View** (Figure 8), we can see that elbow flexion and shoulder abduction synchronization co-occurs on this FMA item in seven stroke individuals (tasks $T_{4}$ and $T_{5}$). This indicates the intrusion of an abnormal flexion synergy when the arm is flexed on seven stroke patients ($b_{1}$ and $b_{2}$). As an example, we can analyze the details of this relationship (task $T_{8}$) in the motion curves of individual “stroke_4” (score 0 out off 2 on “Shoulder Flexion $90^{\circ }$ to $180^{\circ }$” and a total score 27 out of 66 on the FMA) in this component. Specifically, in Figure 8 $b_{4}$, the elbow flexion and shoulder abduction angles are initially stable when the individual lifts the arm, but then there is an increase in the two angles, considering their respective scales, as the arm is fully lifted above the head (task $T_{7}$). This synchronous increase in elbow flexion and shoulder abduction suggests the manifestation of intrusive flexion synergies that can be seen in stroke patients lifting the arm weight against gravity. Moreover, we can see in the scatter plot ($b_{2}$) the distribution according to the mean value (selected in $b_{3}$) of the time series for the selected edge. Notably, as abduction increases, elbow flexion also increases. This increased elbow flexion (despite instructions to keep the elbow straight) implies the intrusion of a flexion synergy.

### 8.3. Experts Comments

Reduced impairment, as evidenced by the re-emergence of normal movement patterns including the absence of intrusive synergies, is the signpost of true motor recovery. Impairment limits activity performance, whereas compensation helps achieve it. Although compensatory motions support functionality in the short-term, they blunt beneficial training effects and increase aberrant plasticity in lesioned animals [52,53]. In humans, preventing compensatory motions during training promotes greater reduction in UE impairment [54]. If the presence and degree of impairment and compensation can be identified in a moving patient, treatment strategies can be adjusted to maximally target impairment and minimize compensation.

With knowledge about impairment and compensation occurring in functional movement, one can generate rules for changing a treatment strategy in real time. For example, if compensatory motions start to escalate, a therapist can downgrade task difficulty to lessen compensation and focus on impairment reduction. The future of health care delivery is also unclear: access to inpatient rehabilitation services is decreasing nationally, with a push toward rehabilitation in the home. Given the growing interest in remote monitoring and training by telerehabilitation, the automated capture and quantitation of movement abnormalities could assist remote rehabilitation training.

The visualization tool, though preliminary, may help identify both impairment and compensation in quasi real-time. This visualization could guide the targeting of impairment and compensation in certain limb segments, facilitating the delivery of personalized rehabilitation interventions.

## 9. Discussion and Limitations

NE-Motion was developed to address a particular application; therefore, it bears some limitations. For instance, NE-Motion might not be appropriated to applications involving a large number of vertices, and some visualization components are not promptly scalable. In fact, the adaptation of NE-Motion to applications involving data sets with hundreds of data streams would demand the redesign of important visualization components, such as the **Pairwise Relations View**.

We believe, though, that the graph learning network representation proposed in this work can serve other scenarios. Applications such as rehabilitation of sports injuries and athletic performance analysis are two examples, as those applications involve the use of sensors to assess and compare individuals and groups of individuals.

As future work, we intend to extend NE-Motion to applications involving multivariate time series databases, as, for example, electroencephalography (EEG).

## 10. Conclusions

In this work, we introduced a visual analytics tool to assist in the rehabilitation process of stroke patients. NE-Motion was developed in close collaboration with domain experts, who helped us to translated their analytical needs into the visualization system. We also proposed a graph learning methodology to represent the movements by the joint angles as a set of networks. A solid mathematical framework to filter the set of networks was also proposed. NE-Motion was validated in two case studies using real data and with feedback from the domain experts, which attested to its effectiveness in addressing the analytical tasks.

## Figures and Tables

**Table 1:** Summary of the main characteristics of existing visualization assisted motion capture data analysis. From left to right, the first column contains a reference to the technique, the second column describes the type of motion data handled by each method, the third column highlights the mathematical and computational model each technique relies on, and the right most column shows the visual metaphors employed in the visualization. Notice that NE-Motion differs from other approaches in all aspects: type of data, modeling and visual metaphor.

Work	Motion Data Type	Modeling Approach	Visual Metaphor
Bernard et al. [12] tool	Common human body movements	Supervised and unsupervised learning	Stick-man glyph and labeled horizontal bars
Krekel et al. [13] tool	Movements performed by patients with fractured proximal humerus	Spline and dimensionality reduction	3-D Human pose view, parallel coordinates, and scatter plot
MotionBrowser [14]	Movements performed by obstetrical brachial plexus patients	Entropy, Kullback–Leibler divergence, and K-means clustering	Curve-charts, histogram and video-replay
Nguyen et al. [15] tool	Patellofemoral joint motion on the knee of operated patients	GPU-based feature identification and tracking technique using SIFT	Radial plot and static 3D sweep plot with magic mirror strategy
KAVAGait [16]	Walk performed by gait abnormalities patients	Fisher’s statistical indicators and filtering strategies	Curve-charts, twin box plots and range sliders
FuryExplorer [17]	Trotting motion of lame horses	Dimensionality reduction and K–D tree clustering	Horse glyphs, projections and multi-curve charts
MotionExplorer [18]	Common human body movements	Divisive hierarchical clustering algorithm	Stick-man glyphs, and tree and directed graphs
GestureAnalyser [19]	Gestures performed with the UE	Interactive agglomerative hierarchical clustering and dynamic time warping	Colored tree graphs and animated UE glyphs
Motionflow [20]	Gestures performed with the UE	Interactive partition-based clustering	Colored tree graphs, treemap and animated UE glyphs
NE-Motion (proposed)	Movements performed by stroke patients using UE	Graph learning, dimensionality reduction and filtering strategies	Arc Visualization, projections, and curve-charts

**Table 2:** List of anatomical angles. The system uses a rigid-body skeletal model to convert the IMU measurements into joint and segment angles. Shoulder total flexion is a combination of shoulder flexion/extension and shoulder ad-/abduction. Thoracic angles are computed between the cervical vertebra and the thoracic vertebra. Lumbar angles are computed between the thoracic vertebra and pelvis.

Joint/Segment	Anatomical Angle
Shoulder	Shoulder flexion/extension Shoulder internal/external rotation Shoulder ad-/abduction Shoulder total flexion
Elbow	Elbow flexion/extension
Wrist	Wrist flexion/extension Forearm pronation/supination Wrist radial/ulnar deviation
Thorax	Thoracic flexion/extension Thoracic axial rotation Thoracic lateral flexion/extension
Lumbar	Lumbar flexion/extension Lumbar axial rotation Lumbar lateral flexion/extension

**Table 3:** Demographic characteristics of patients in the data set. The range (between parenthesis) and average values are shown for the age, years since stroke, and FMA score.

Demographic Property	Stroke	Control
Number of patients	51	18
Age (years)	57.76 (21.26–84.26)	61.55 (41.99–82.96)
Male	23	10
Female	28	8
Ischemic Stroke	42	-
Hemorrhagic Stroke	9	-
Years since stroke	5.34 (0.26–38.44)	-
Paretic side	28 left and 23 right	-
FMA score (points)	43.11 (8–65)	65.475 (62–66)

## Data Availability

The dataset analyzed for the current study are available from the authors on reasonable request.

## Abbreviations

The following abbreviations are used in this manuscript:

AE	Autoencoder
CTRL	Control
EEG	Electroencephalography
FMA	Fugl–Meyer Assessment
FPS	Frames Per Second
GL	Graph Learning
ID	Identification Code
IMU	Inertial Measurement Unit
LE	Lower Extremity
MDS	Multidimensional Scaling
NE-Motion	Network Environment for Motion Capture Data Analysis
PCA	Principal Component Analysis
UE	Upper Extremity
WSNs	Wearable Sensors Networks

## References

[1] Warlow C.P., Van Gijn J., Dennis M.S., Wardlaw J.M., Bamford J.M., Hankey G.J., Sandercock P.A., Rinkel G., Langhorne P., Sudlow C. (2011). Stroke: Practical Management.

[2] Bonita R., Mendis S., Truelsen T., Bogousslavsky J., Toole J., Yatsu F. (2004). The global stroke initiative. Lancet Neurol..

[3] Fugl-Meyer A.R., Jääskö L., Leyman I., Olsson S., Steglind S. (1975). The post-stroke hemiplegic patient. 1. A method for evaluation of physical performance. Scand. J. Rehabil. Med..

[4] Del Din S., Patel S., Cobelli C., Bonato P. Estimating Fugl-Meyer clinical scores in stroke survivors using wearable sensors. Proceedings of the 2011 Annual International Conference of the IEEE Engineering in Medicine and Biology Society.

[5] Wang J., Yu L., Wang J., Guo L., Gu X., Fang Q. Automated Fugl-Meyer assessment using SVR model. Proceedings of the 2014 IEEE International Symposium on Bioelectronics and Bioinformatics (IEEE ISBB 2014).

[6] Yu L., Xiong D., Guo L., Wang J. (2016). A remote quantitative Fugl-Meyer assessment framework for stroke patients based on wearable sensor networks. Comput. Methods Programs Biomed..

[7] Olson J., Redkar S. (2018). A survey of wearable sensor networks in health and entertainment. MOJ Appl. Bionics Biomech..

[8] Ranganathan R., Wang R., Dong B., Biswas S. (2017). Identifying compensatory movement patterns in the upper extremity using a wearable sensor system. Physiol. Meas..

[9] Khan R.A., Pathan A.S.K. (2018). The state-of-the-art wireless body area sensor networks: A survey. Int. J. Distrib. Sens. Netw..

[10] Mosenia A., Sur-Kolay S., Raghunathan A., Jha N.K. (2017). Wearable Medical Sensor-Based System Design: A Survey. IEEE Trans. Multi-Scale Comput. Syst..

[11] Schroeder D., Korsakov F., Knipe C.M.P., Thorson L., Ellingson A.M., Nuckley D., Carlis J., Keefe D.F. (2014). Trend-centric motion visualization: Designing and applying a new strategy for analyzing scientific motion collections. IEEE Trans. Vis. Comput. Graph..

[12] Bernard J., Dobermann E., Vögele A., Krüger B., Kohlhammer J., Fellner D. (2017). Visual-interactive semi-supervised labeling of human motion capture data. Electron. Imaging.

[13] Krekel P.R., Valstar E.R., De Groot J., Post F.H., Nelissen R.G., Botha C.P. (2010). Visual Analysis of Multi-Joint Kinematic Data. Comput. Graph. Forum.

[14] Chan G.Y.Y., Nonato L.G., Chu A., Raghavan P., Aluru V., Silva C.T. (2019). Motion Browser: Visualizing and Understanding Complex Upper Limb Movement Under Obstetrical Brachial Plexus Injuries. IEEE Trans. Vis. Comput. Graph..

[15] Nguyen K.T., Gauffin H., Ynnerman A., Ropinski T. (2016). Quantitative Analysis of Knee Movement Patterns Through Comparative Visualization. Visualization in Medicine and Life Sciences III.

[16] Wagner M., Slijepcevic D., Horsak B., Rind A., Zeppelzauer M., Aigner W. (2018). KAVAGait: Knowledge-assisted visual analytics for clinical gait analysis. IEEE Trans. Vis. Comput. Graph..

[17] Wilhelm N., Vögele A., Zsoldos R., Licka T., Krüger B., Bernard J. (2015). Furyexplorer: Visual-interactive exploration of horse motion capture data. Visualization and Data Analysis 2015.

[18] Bernard J., Wilhelm N., Krüger B., May T., Schreck T., Kohlhammer J. (2013). Motionexplorer: Exploratory search in human motion capture data based on hierarchical aggregation. IEEE Trans. Vis. Comput. Graph..

[19] Jang S., Elmqvist N., Ramani K. GestureAnalyzer: Visual analytics for pattern analysis of mid-air hand gestures. Proceedings of the 2nd ACM Symposium on Spatial User Interaction.

[20] Jang S., Elmqvist N., Ramani K. (2015). Motionflow: Visual abstraction and aggregation of sequential patterns in human motion tracking data. IEEE Trans. Vis. Comput. Graph..

[21] Aigner W., Miksch S., Müller W., Schumann H., Tominski C. (2007). Visualizing time-oriented data—A systematic view. Comput. Graph..

[22] Keim D.A., Nietzschmann T., Schelwies N., Schneidewind J., Schreck T., Ziegler H. A spectral visualization system for analyzing financial time series data. Proceedings of the Eurographics/IEEE TCVG Symposium on Visualization.

[23] Zhao J., Chevalier F., Pietriga E., Balakrishnan R. (2011). Exploratory analysis of time-series with chronolenses. IEEE Trans. Vis. Comput. Graph..

[24] Ward M.O., Guo Z. (2011). Visual Exploration of Time-Series Data with Shape Space Projections. Comput. Graph. Forum..

[25] Bernard J., Hutter M., Reinemuth H., Pfeifer H., Bors C., Kohlhammer J. (2019). Visual-Interactive Preprocessing of Multivariate Time Series Data. Comput. Graph. Forum..

[26] Doleisch H., Gasser M., Hauser H. (2003). Interactive feature specification for focus+ context visualization of complex simulation data. VisSym.

[27] Kehrer J., Ladstädter F., Muigg P., Doleisch H., Steiner A., Hauser H. (2008). Hypothesis generation in climate research with interactive visual data exploration. IEEE Trans. Vis. Comput. Graph..

[28] Poco J., Dasgupta A., Wei Y., Hargrove W., Schwalm C., Cook R., Bertini E., Silva C. (2014). SimilarityExplorer: A Visual Inter-Comparison Tool for Multifaceted Climate Data. Comput. Graph. Forum.

[29] Julien Y., Sobrino J.A. (2021). Introducing the Time Series Change Visualization and Interpretation (TSCVI) method for the interpretation of global NDVI changes. Int. J. Appl. Earth Obs. Geoinf..

[30] Li Q., Xu P., Chan Y.Y., Wang Y., Wang Z., Qu H., Ma X. (2016). A visual analytics approach for understanding reasons behind snowballing and comeback in moba games. IEEE Trans. Vis. Comput. Graph..

[31] Valdivia P., Dias F., Petronetto F., Silva C.T., Nonato L.G. Wavelet-based visualization of time-varying data on graphs. Proceedings of the 2015 IEEE Conference on Visual Analytics Science and Technology (VAST).

[32] Dal Col A., Valdivia P., Petronetto F., Dias F., Silva C.T., Nonato L.G. (2017). Wavelet-based visual analysis of dynamic networks. IEEE Trans. Vis. Comput. Graph..

[33] Zanabria G.G., Silveira J.A., Poco J., Paiva A., Nery M.B., Silva C.T., de Abreu S.F.A., Nonato L.G. (2020). CrimAnalyzer: Understanding Crime Patterns in São Paulo. IEEE Trans. Vis. Comput. Graph..

[34] Chen W., Guo F., Wang F.Y. (2015). A survey of traffic data visualization. IEEE Trans. Intell. Transp. Syst..

[35] Kehrer J., Hauser H. (2012). Visualization and visual analysis of multifaceted scientific data: A survey. IEEE Trans. Vis. Comput. Graph..

[36] Goodyear M.D., Krleza-Jeric K., Lemmens T. (2007). The Declaration of Helsinki.

[37] Williams J.R. (2008). The Declaration of Helsinki and public health. Bull. World Health Organ..

[38] Averbuch A.Z., Neittaanmaki P., Zheludev V.A. (2014). Spline and Spline Wavelet Methods with Applications to Signal and Image Processing: Volume I: Periodic Splines.

[39] Dong X., Thanou D., Rabbat M., Frossard P. (2019). Learning graphs from data: A signal representation perspective. IEEE Signal Process. Mag..

[40] Zosso D., Osting B., Osher S.J. A dirichlet energy criterion for graph-based image segmentation. Proceedings of the 2015 IEEE International Conference on Data Mining Workshop (ICDMW).

[41] Kalofolias V. (2016). How to learn a graph from smooth signals. Artificial Intelligence and Statistics.

[42] Kalofolias V., Perraudin N. (2017). Large scale graph learning from smooth signals. arXiv.

[43] Komodakis N., Pesquet J.C. (2015). Playing with duality: An overview of recent primal-dual approaches for solving large-scale optimization problems. IEEE Signal Process. Mag..

[44] Correa R., Jofré A., Thibault L. (1992). Characterization of Lower Semicontinuous Convex Functions. Proc. Am. Math. Soc..

[45] Jech T. (2013). Set Theory.

[46] Wattenberg M. Arc diagrams: Visualizing structure in strings. Proceedings of the IEEE Symposium on Information Visualization.

[47] Opsahl T., Agneessens F., Skvoretz J. (2010). Node centrality in weighted networks: Generalizing degree and shortest paths. Soc. Netw..

[48] Brandes U. (2001). A faster algorithm for betweenness centrality. J. Math. Sociol..

[49] Berkhin P. (2005). A survey on pagerank computing. Internet Math..

[50] Watts D.J., Strogatz S.H. (1998). Collective dynamics of ‘small-world’networks. Nature.

[51] Defferrard M., Martin L., Pena R., Perraudin N. (2017). PyGSP: Graph Signal Processing in Python. Zenodo.

[52] Allred R., Maldonado M., Jones T. (2005). Training the “less-affected” forelimb after unilateral cortical infarcts interferes with functional recovery of the impaired forelimb in rats. Restor. Neurol. Neurosci..

[53] Kim S.Y., Allred R.P., Adkins D.L., Tennant K.A., Donlan N.A., Kleim J.A., Jones T.A. (2015). Experience with the “good” limb induces aberrant synaptic plasticity in the perilesion cortex after stroke. J. Neurosci..

[54] Michaelsen S.M., Dannenbaum R., Levin M.F. (2006). Task-specific training with trunk restraint on arm recovery in stroke: Randomized control trial. Stroke.

